# Unveiling the Uncommon: A Case of Eosinophilic Granulomatosis With Polyangiitis Featuring Atypical Presentation and Diagnostic Challenges

**DOI:** 10.7759/cureus.72564

**Published:** 2024-10-28

**Authors:** Diogo Macedo, Ana Gonçalves, Ana Mafalda Pais, Ana Silva, Ana Clara Coelho, Rute Cacola

**Affiliations:** 1 Internal Medicine, Unidade Local de Saúde Gaia Espinho, Vila Nova de Gaia, PRT; 2 Physical Medicine and Rehabilitation, Unidade Local de Saúde Gaia Espinho, Vila Nova de Gaia, PRT; 3 Pulmonology, Unidade Local de Saúde Gaia Espinho, Vila Nova de Gaia, PRT

**Keywords:** anca-associated vasculitis, differential for fever of unknown origin, eosinophilic granulomatosis with polyangiitis (egpa), mononeuropathy multiplex, mpo-anca, peripheral eosinophilia, quality of life (qol)

## Abstract

Eosinophilic granulomatosis with polyangiitis (EGPA) is a rare necrotizing vasculitis affecting small- to medium-sized vessels that can manifest in a multisystemic manner. While the classic triad of rhinosinusitis, asthma, and eosinophilia is commonly associated, it is essential to recognize that these features are not mandatory for diagnosis.

We present a case of a 61-year-old woman with a unique EGPA manifestation who was hospitalized because of a seven-month history of weight loss, asthenia, epigastric abdominal pain, peripheral eosinophilia, and paresthesia in the left feet and hand. Notably absent were typical pulmonary alterations such as asthma or nasal polyps. Respiratory function tests yielded normal results, and imaging studies revealed no granulomas or signs of vasculitis. Instead, she exhibited mononeuritis multiplex and constitutional symptoms. Diagnostic criteria were met, including positive anti-neutrophil cytoplasmic antibody (ANCA) with myeloperoxidase (MPO) pattern and severe axonal neuropathy confirmed by nerve biopsy. Treatment commenced with prednisolone and then with methotrexate.

This case underscores the diversity of EGPA presentations and the importance of considering atypical symptoms for accurate diagnosis and timely intervention. Despite the absence of classic features, our comprehensive approach facilitated prompt recognition and effective management.

## Introduction

Eosinophilic granulomatosis with polyangiitis (EGPA) is a rare necrotizing vasculitis affecting small- to medium-sized vessels, capable of presenting in a multisystemic manner [1]. Although common manifestations of EGPA include chronic rhinosinusitis, asthma, and marked peripheral blood eosinophilia, it is imperative to recognize that these clinical features are not mandatory for diagnostic consideration. Within the spectrum of positive anti-neutrophil cytoplasmic antibody (ANCA) vasculitis, namely, EGPA, granulomatosis with polyangiitis (GPA), and microscopic polyangiitis, EGPA represents the least prevalent [2]. The annual incidence is estimated to range from 0.9 to 2.4 cases per million individuals, with a heightened predisposition observed in individuals aged 40-60 years [3].

EGPA typically unfolds in three discernible stages. The prodromal phase, manifesting in the second or third decade of life, is characterized by allergic rhinitis and asthma. The eosinophilic stage entails the infiltration of eosinophils into various tissues and organs, particularly the lungs and gastrointestinal tract. The concluding phase is the vasculitic stage, marked by life-threatening systemic vasculitis affecting small- and medium-sized vessels, featuring granulomatosis, constitutional symptoms, and peripheral neuropathy [4-6]. Notably, these phases may exhibit overlap.

This case report delineates a distinct and atypical presentation of EGPA, thereby underscoring the clinical heterogeneity inherent in the disease.

## Case presentation

A 61-year-old nonsmoking woman with a medical history of Hashimoto's hypothyroidism, plantar fasciitis, and depression presented to the emergency department (ED) with a seven-month history of weight loss (17 kg), asthenia, epigastric pain, reduced dietary tolerance, paresthesia in the left extremities, and peripheral eosinophilia. She denied symptoms such as dysphagia, nausea, vomiting, gastrointestinal bleeding, or any noticeable changes in stool pattern or color. Furthermore, she reported no recent travel history, owned no pets, and resided in a well-maintained household. Having previously sought medical attention for peripheral eosinophilia, she had undergone treatment with albendazole, yielding no resolution of the issue. Upon ED evaluation, the physical examination revealed no specific abnormalities, except for tenderness upon deep palpation in the epigastric region. Laboratory analysis demonstrated peripheral eosinophilia (3090/μL) and an elevated C-reactive protein (CRP) level of 19.53 mg/dL with no further alterations. Urinalysis did not reveal any significant abnormalities. In light of these findings, an abdominal-pelvic computed tomography (CT) scan was performed, revealing diffuse alterations in both kidneys suggestive of bilateral pyelonephritis. Given the unexplained eosinophilia and the observed renal findings, the patient was subsequently admitted to the internal medicine department and initiated on ceftriaxone therapy.

During the patient's hospitalization, she developed refractory fever with negative blood and urine cultures. A nine-day course of ceftriaxone failed to alleviate her symptoms. Due to persistent eosinophilia, a single dose of ivermectin was administered without improvement. Subsequently, a bronchoscopy revealed a bronchoalveolar lavage without eosinophilia. Fecal parasitology (*Giardia lamblia* and *Cryptosporidium*), parasitic serology (bilharzia, *Fasciola*, *Strongyloides*, and *Trichinella*), and *Coxiella burnetii* yielded negative results.

Physical examination continued to reveal tenderness in the right abdominal quadrant, hypoesthesia on the left side of the body, and paresis in wrist flexion, finger flexion, and thumb opposition of the left hand. Neurological consultation reported paresis grade 4+ in specific upper extremity movements, with normal strength in other segments. Sensory deficits included hypoesthesia in the palmar region of the left hand and feet, with hyperesthesia observed in specific areas. Deep tendon reflexes were globally grade 2, and there were bilateral flexor plantar responses. Gabapentin was initiated for neuropathic pain.

Laboratory analyses showed leukocytosis with hypereosinophilia, elevated IgE (373.00 IKU/L), elevated C-reactive protein (16.49 mg/dL), and elevated sedimentation velocity (84 mm/H1). Positive anti-neutrophil cytoplasmic antibody (ANCA) (1/320) with a myeloperoxidase (MPO) pattern (155 RU/mL) was also noted (Table 1).

**Table 1:** Laboratory values ANCA, anti-neutrophil cytoplasmic antibody; MPO, myeloperoxidase; P-ANCA, perinuclear anti-neutrophil cytoplasmic antibody

Parameters	Values
Erythrocyte sedimentation rate	84 mm/H1
Leukocytes	16.03×10^3^/UL
Eosinophils	3090/μL
IgE	373.00 IKU/L
ANCA	1/320 (P-ANCA pattern)
ANCA-MPO	155 RU/mL
C-reactive protein	16.49 mg/dL

**Figure 1:**
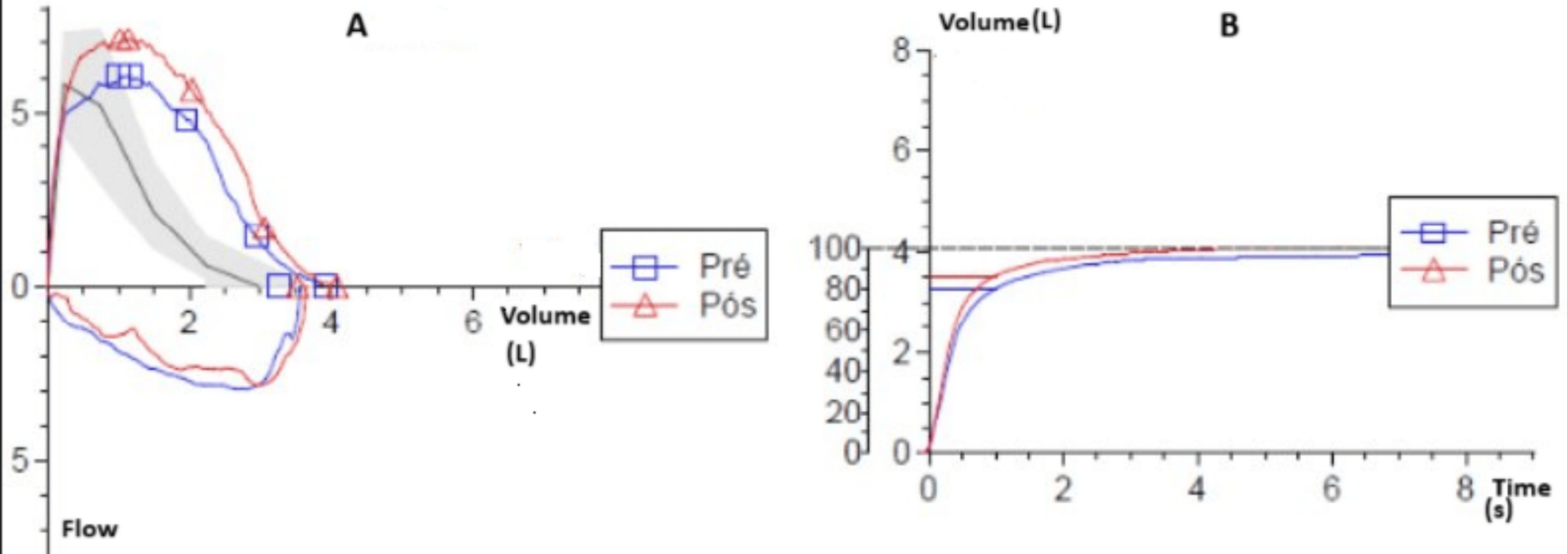
Pulmonary function test results showing flow-volume and volume-time curves Panel A: Flow-volume curve comparing pre-bronchodilator (blue line, labeled "Pré") and post-bronchodilator (red line, labeled "Pós") measurements. The x-axis represents the volume of air expired in liters (L), and the y-axis indicates the flow rate in liters per second (L/s). The shaded area represents the normal reference range. The similarity in the shape and peak flow values of the two curves suggests minimal change in airway resistance after bronchodilator administration, indicating a negative bronchodilation response. Panel B: Volume-time curve comparing pre-bronchodilator (blue line) and post-bronchodilator (red line) measurements. The x-axis represents time in seconds (s), while the y-axis shows the cumulative volume of air expired in liters (L). Both curves reach a plateau at similar volumes, indicating no significant change in forced vital capacity (FVC) and forced expiratory volume in the first second (FEV1) following bronchodilator use. This further confirms a negative bronchodilator response, suggesting the absence of reversible airway obstruction

**Figure 2:**
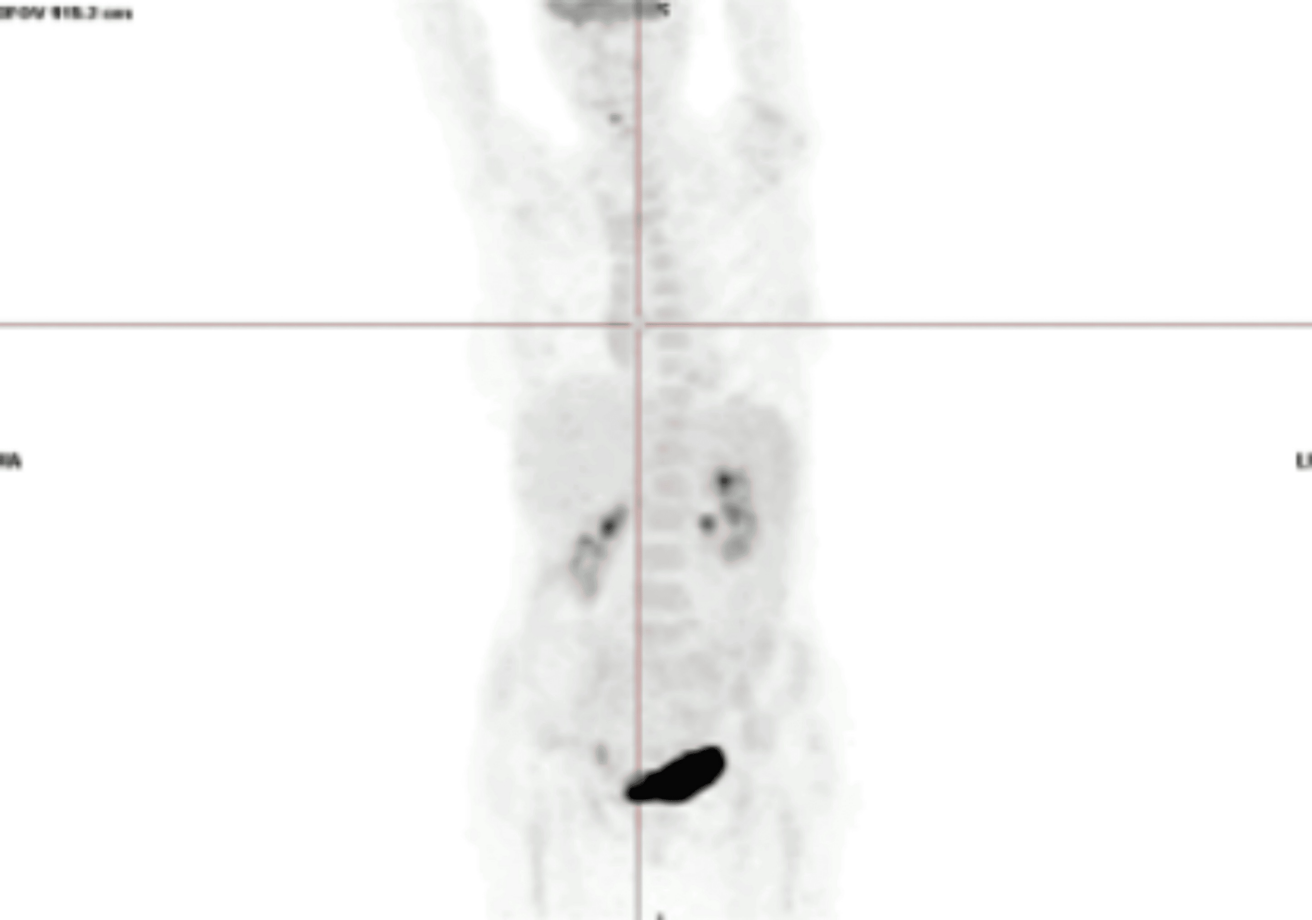
Total body PET-CT Demonstrated nonspecific 18F-FDG uptake in the cervical lymph nodes, spleen, and epiglottic fat, without any specific lesions CT, computed tomography; 18F-FDG, 18F-fluorodeoxyglucose

Imaging studies, including respiratory function tests (Figure 1), CT of the paranasal sinus, and CT imaging of the neck, chest, abdomen, and pelvis, revealed no granulomas or signs of vasculitis. Transesophageal echocardiography and cardiac MRI ruled out vegetations but showed an inflammation of the pericardium and myocardium. The MRI showed hypokinesia of the distal segment of the interventricular septum and multiple areas of hypersignal in T2 and delayed enhancement after gadolinium with subepicardial/intramural distribution in the basal segment of the inferolateral wall and subendocardial distribution in the distal segments of the interventricular septum and midsegment of the inferolateral wall, not following a coronary irrigation territory. Endoscopic studies were unremarkable. Bone marrow biopsy showed no significant alterations, while a total body PET-CT demonstrated nonspecific 18F-fluorodeoxyglucose (18F-FDG) uptake in the cervical lymph nodes, spleen, and epiglottic fat (Figure 2). Electromyography results indicated mononeuritis multiplex affecting various nerves with axonal neuropathy in the superficial and deep peroneal, tibial, and bilateral sural nerves in the lower limbs and in the upper limbs with the involvement of axonal and myelinic neuropathy in the ulnar and median nerves (Table 2).

**Table 2:** Electromyography (EMG) result interpretation EMG showing mononeuritis multiplex affecting various nerves with axonal neuropathy in the superficial and deep peroneal, tibial, and bilateral sural nerves in the lower limbs and in the upper limbs with the involvement of axonal and myelinic neuropathy in the ulnar and median nerves Amplitude: +, mildly increased amplitude; ++, moderately increased amplitude. Duration: +, mildly prolonged duration; ++, moderately prolonged duration. Polyphasia: +, mild polyphasia; ++, moderate polyphasia Fib, fibrillation; PSW, positive sharp waves; Fasc, fasciculation; Amp, amplitude; Poly, polyphasia

Muscle	Interpretation	Fib (0/10)	PSW (0/10)	Fasc	Amp (voluntary activity)	Duration (voluntary activity)	Poly (voluntary activity)	Stability (voluntary activity)
Right interosseous dorsal I	Normal	0/10	0/10	0	Normal	Normal	Normal	Normal
Left interosseous dorsal I	Normal	0/10	0/10	0	Normal	Normal	Normal	Normal
Right extensor digitorum communis	Normal	0/10	0/10	0	+	+	Normal	Jitter
Left extensor digitorum communis	Slight subacute neuropathy	0/10	0/10	0	+	Normal	+	Jitter
Right flexor carpi radialis	Normal	0/10	0/10	0	Normal	Normal	Normal	Normal
Left flexor carpi radialis	Slight subacute neuropathy	0/10	0/10	0	+	Normal	+	Jitter
Right biceps	Normal	0/10	0/10	0	Normal	Normal	Normal	Normal
Left biceps	Normal	0/10	0/10	0	+	Normal	+	Jitter
Right deltoid anterior	Normal	0/10	0/10	0	+	Normal	Normal	Jitter
Left deltoid anterior	Slight subacute neuropathy	0/10	0/10	0	+	+	Normal	Jitter
Right abductor hallucis	Moderate subacute neuropathy	6/10	6/10	0	++	Normal	++	Jitter
Left abductor hallucis	Slight subacute neuropathy	4/10	4/10	0	++	Normal	+	Jitter
Right gastrocnemius medialis	Moderate subacute neuropathy	4/10	4/10	0	++	+	++	Jitter
Left gastrocnemius medialis	Slight subacute neuropathy	4/10	4/10	0	++	+	++	Jitter
Right peroneus longus	Slight subacute neuropathy	4/10	4/10	0	+	+	++	Jitter
Left peroneus longus	Normal	0/10	0/10	0	Normal	Normal	Normal	Normal
Right tibialis anterior	Slight subacute neuropathy	0/10	0/10	0	Normal	Normal	Normal	Normal
Left tibialis anterior	Normal	0/10	0/10	0	Normal	Normal	Normal	Normal
Right vastus lateralis	Normal	0/10	0/10	0	Normal	Normal	Normal	Normal
Left vastus lateralis	Normal	0/10	0/10	0	Normal	Normal	Normal	Normal

Given the persistent symptoms, prednisolone at 1 mg/kg/day was initiated, resulting in the resolution of eosinophilia, fever, and reduced epigastric pain within two days. Some minor recovery of nerves affected by mononeuropathy multiplex was observed.

The patient was discharged on the 16th day of prednisolone therapy, exhibiting afebrile status, resolved eosinophilia, the absence of epigastric pain, and enhanced food tolerance. Subsequently, she received a referral to the internal medicine-autoimmune disease consult and underwent a nerve biopsy, which confirmed an actively severe axonal neuropathy with a vasculitic etiology. Satisfying the EGPA criteria as per the 2022 American College of Rheumatology, she accrued six points in this classification, the presence of mononeuropathy multiplex (one point) and blood eosinophil count bigger than 1×10E^9^/L. Following the confirmation of the diagnosis, the patient's therapeutic regimen included prednisolone at an initial dose of 1 mg/kg/day, gradually tapered to 2.5 mg daily. Additionally, methotrexate at a weekly dose of 12.5 mg was introduced, resulting in effective symptom control.

Following a thorough reassessment and deliberation within the internal medicine-autoimmune disease consult, considering the current symptomatic control of the patient, it has been decided not to initiate mepolizumab treatment. Additionally, a new electromyography was performed, which demonstrated overall neurological improvement, with an increased amplitude of response in the evaluated nerves.

## Discussion

Eosinophilic granulomatosis with polyangiitis (EGPA) is an infrequent subtype of ANCA-MPO-positive vasculitis, renowned for its varied clinical manifestations. This case underscores an unconventional presentation of EGPA, marked by the absence of typical pulmonary or renal involvement and the presence of peripheral eosinophilia, abdominal pain, mononeuropathy multiplex, and constitutional symptoms. Given the departure from the customary clinical profile, an extensive diagnostic approach was undertaken to eliminate alternative etiologies. Initial investigations primarily focused on excluding potential causes of eosinophilia, encompassing bacterial or parasitic infections, as well as autoimmune disorders. Subsequent to this, a comprehensive imaging study of the entire body was conducted to rule out malignancy and other infectious and inflammatory conditions.

While mononeuropathy multiplex is not the most prevalent symptom associated with EGPA, it manifests in approximately 75% of cases and is more frequent in ANCA-MPO-positive EGPA compared to cases where these antibodies are negative. Typically, it presents with pain, numbness, and/or weakness in at least two distinct nerve areas and may progress to a symmetric or asymmetric polyneuropathy if left untreated [7-10]. In this specific case, the patient exhibited paresis and hyperesthesia in precise upper extremity movements, coupled with hypoesthesia in the lower extremities, impacting her ambulatory capacity and daily activities.

Formulating a singular algorithm for evaluating the possibility of vasculitis proves challenging due to the extensive range of potential symptoms. Nonetheless, certain clinical and laboratory parameters may guide toward this diagnosis. In this case, constitutional symptoms, multiorgan/system involvement, the presence of ANCA-positive antibodies, eosinophilia, and mononeuropathy multiplex were pivotal in considering vasculitis. Subsequently, confirmation via nerve biopsy revealed vasculitis as the probable diagnosis. Although the typical symptomatology was not entirely evident, an assessment based on the 2022 American College of Rheumatology/European Alliance of Associations for Rheumatology classification criteria for EGPA indicated a score of six points, equalizing the required six points for EGPA classification [11].

The cornerstone of remission treatment for EGPA vasculitis is high-dose glucocorticoids, particularly prednisone at 0.5-1 mg/kg/day. Higher doses are typically reserved for severe manifestations, such as cardiac involvement or neuropathy [12,13]. In this case, given the presentation of mononeuropathy multiplex, high-dose prednisone was initiated and subsequently tapered. Additionally, methotrexate was introduced during the remission induction, a choice often made in non-severe EGPA vasculitis due to its lower toxicity compared to rituximab or cyclophosphamide [13]. Over the initial seven months of treatment, the patient exhibited substantial improvement, with the resolution of abdominal pain, gastrointestinal symptoms, and weight recovery.

Further, an electromyography study further substantiates the patient's improvement, particularly in terms of nerve function. Motor conduction studies indicate that compound muscle action potentials (CMAPs) vary across nerves, showing low to very low amplitudes in some (left median, left peroneal, and right tibial) while normal amplitudes in others (right median, bilateral ulnar, right peroneal, and left tibial). Motor nerve conduction velocities remain within normal limits or are slightly reduced, aligning proportionately with any amplitude decreases. Sensory conduction studies reveal normal amplitudes in sensory nerve action potentials (SNAPs) in some nerves (bilateral radial and bilateral ulnar) and low amplitudes in others (bilateral median, bilateral superficial peroneal, and bilateral sural). Sensory nerve conduction velocities are similarly normal or proportionately reduced where the amplitude is lower. Compared to the prior assessment on May 8, 2023, there has been a global improvement in both CMAP and SNAP amplitudes, with significant gains, including a doubling of the left median CMAP and a tripling of the right tibial CMAP amplitude. These findings suggest ongoing recovery from the mononeuropathy multiplex leading to restored ambulation without assistance or claudication and the amelioration of paresis. However, residual paresthesias persisted in the lower limbs, albeit with diminished intensity compared to the initial presentation. Although mepolizumab has shown efficacy in various case reports and studies for diminishing peripheral neuropathy and improving nerve conduction velocity, addressing the most intense and morbidity-inducing symptoms in this patient, due to the improvement in symptoms demonstrated by the patient, along with the improvement shown in the subsequent electromyography, the patient will, for now, remain on a tapering regimen of prednisolone and methotrexate, without transitioning to mepolizumab (Table 3) [14,15].

**Table 3:** Laboratory values after seven months of targeted treatment This table shows the laboratory values presented by the patient after therapy with corticosteroids and methotrexate. Here, we can see that there was a normalization of almost all values, especially the erythrocyte sedimentation rate and the C-reactive protein, which are important markers of inflammation

Parameters	Values
Erythrocyte sedimentation rate	5 mm/H1
Leukocytes	12.00x10^3^/UL
Eosinophils	160/μL
IgE	230.00 IKU/L
C-reactive protein	0.19 mg/dL

## Conclusions

In conclusion, this case report emphasizes that, akin to other vasculitic diseases, EGPA can present with a diverse array of symptoms. Even in the absence of common symptoms such as pulmonary involvement, the diagnosis can still be established. In this case, atypical symptoms such as prolonged weight loss, epigastric pain, peripheral eosinophilia, and mononeuropathy multiplex were prominent features. These less typical but significant manifestations underscore the importance of maintaining a high index of suspicion and conducting a thorough evaluation in challenging cases. By recognizing these atypical symptoms and utilizing a comprehensive diagnostic approach, we successfully established a diagnosis and commenced treatment. Following the initiation of treatment with prednisolone and methotrexate, the patient showed significant improvement in her symptoms, including reduced pain, recovery of weight, and overall better physical function. Furthermore, her analytical results showed marked improvement, and follow-up EMG studies revealed enhanced nerve conduction, with increased amplitude in both motor and sensory potentials. This comprehensive approach not only facilitated the accurate diagnosis but also led to significant improvement in the patient's quality of life.
